# Rewarding outcomes enhance attentional capture and delay attentional disengagement

**DOI:** 10.7717/peerj.15868

**Published:** 2023-08-18

**Authors:** Minmin Yan, Zong Meng, Na Hu, Antao Chen

**Affiliations:** 1 Faculty of Psychology, Southwest University, Chongqing, China; 2 Department of Preschool & Special Education, Kunming University, Kunming, China; 3 School of Psychology, Shanghai University of Sport, Shanghai, China

**Keywords:** Attentional capture, Attentional disengagement, Reward, Loss

## Abstract

Attentional capture and disengagement are distinct process involved in attentional orienting. Most current studies have examined either the process of attentional capture or disengagement by manipulating stimuli associated with either positive (gains) or negative outcomes (losses). However, few studies have investigated whether attentional capture and disengagement are modulated by reward and loss outcomes. In the current study, we want to examine whether positive or negative outcomes could modulate distinguishing process of attentional capture and disengagement. Here, we manipulated different colored singleton stimuli associated with reward or loss outcomes; these stimuli were either presented at the center of screen or at the peripheral location. The participants’ task was to search the target and identify the orientation of line segment in target as quickly as possible. The results showed that people had difficulty disengaging from a central reward-distractor, in comparison to loss- and neutral-distractor when target was presented at peripheral location. Similarly, peripheral reward-distractor captured more attention than loss- and neutral-distractor when target was presented at the center of screen after central fixation disappeared. Through our discoveries, we can conclude that positive rewards can increase attentional capture and delay attentional disengagement in healthy people.

## Introduction

Our visual attention is naturally drawn to salient objects, affective, and value-associated stimuli that we encounter in our daily lives (Anderson & Yantis, 2013; Awh, Belopolsky & Theeuwes, 2012; Gaspelin & Luck, 2018; Gupta, Hur & Lavie, 2016). Once we realize that these stimuli are task-irrelevant, we can direct our attention away from them and focus on the goal. This attentional processes is thought to be mainly caused by two components: attentional engagement (*i.e.,* capture) and disengagement (Bar-Haim et al., 2007; Tsegaye et al., 2020).

In a classic visual search task, one target and five or more distractors (one of distractors was imbued with value) were presented at peripheral location after central fixation disappeared, and participants were required to respond to orientation of line within target and ignore other distractors. The results showed that participants responded to the target slower when a high-reward distractor was presented, compared to when a low-reward distractor was presented or when no distractor was present (Anderson, Laurent & Yantis, 2011; Wentura, Muller & Rothermund, 2014). Researchers concluded that valuable stimuli capture more attention than neutral or distractor-absent stimuli. However, once attention was captured by a valuable distractor, participants may have difficulty disengaging from these evaluable stimuli. Thus, the overall manual response times (RTs) may be an aggregate of both attentional capture and disengagement stages of cognition (Müller, Rothermund & Wentura, 2016b; Zhuang et al., 2021).

The traditional dot-probe tasks are used to measure attentional bias or capture. Participants are shown two cues, one at the left and one at right of the central fixation, for a short time. Usually, one of the cues is tied to a reward value, and the other is neutral. After that, a target (or a dot probe) is randomly placed at one of two cue locations. Participants’ response times should be faster when the target is presented at the value cue location than at neutral cue location, and indicating that participants’ attention has been captured by the value stimuli.

Recently, Müller, Rothermund & Wentura (2016b) used a modified dot-probe paradigm to distinguish the processes between attentional capture and disengagement by using evaluative stimuli. Especially, they manipulated the neutral baseline with a neutral cue to control “behavioral freezing”, which was a non-attention effect and could slowdown individual response. Müller, Rothermund & Wentura (2016b) measured attentional disengagement by comparing the neutral and invalid condition. The invalid condition means a target is presented at the opposite location of the value cue that had previously appeared, whereas valid condition means that a target is presented at the location of value cue. Neutral baseline refers to a target being randomly presented at one of locations where the previous two neutral cue appeared. The results showed that there was a delayed disengagement from the reward cue when comparing the neutral and invalid condition and there was no attentional capture differences between neutral and valid condition, suggesting there was a disengagement effect. However, it is difficult to rule out floor effects in these results. Floor effects could potentially lead to similar outcomes. In other words, there may be a capture effect in valid trials compared to neutral trials (attention is more likely to be biased toward the position of value cue), however, floor effects have prevented this capture effect from being detected (Watson et al., 2020), thus resulting in no capture effect in valid trials. Altogether, the modified dot-probe paradigm may be inadequate in differentiating the processes of attentional capture by valuable stimuli and delayed disengagement from such stimuli.

Thus, we wanted to distinguish attentional capture and disengagement processes, and investigated the effect of evaluative stimuli on these processes. We modified the visual search task based on the experimental design by Watson et al. (2020). The singleton distractor was at central location after fixation disappeared and target and other non-singleton distractors were presented at peripheral location. As a central distractor was already at the center, participants had to shift their attention away from central distractor location to search target, which could minimize the influence of any effect of central distractor on spatial attentional capture (Watson et al., 2020). However, this procedure only measure attention away from central distractor, *i.e.,* attentional disengagement. It is unclear to what extent people engage with the distractor. Thus, we added an attentional capture condition by manipulating target at central location, and a singleton distractor and others non-singleton distractors were at peripheral location. As a target was already at central location after fixation disappeared, it was not necessary to direct attention toward a peripheral distractor. If response time in reward-distractor trials are larger than those in loss/neutral-distractor trials, this would indicate that peripheral reward-distractor capture more attention. Crucially, we used this paradigm to explore whether evaluative distractors capture more attention than neutral distractors, and whether participants would stay longer on evaluative distractors in comparison to neutral distractors.

## Materials & Methods

### Participants

G*Power software (Faul et al., 2007) indicated that a sample of 24 participants allowed for detection of an interaction of value-distractor and distractor-location of medium effect size (*f* = 0.25) with a power of (1 − *β*) = 0.80 (*α* = 0.05). We recruited 30 college students ranging in age from 18 to 24 years (21 females; age: *M* = 21.4 years, SEM = 0.31) from Southwest University. All participants provided written consent and were given monetary compensation for their participation. They reported that they were physically healthy, with no history of neurological or psychiatric disease. The Human Ethics Committee of Southwest University, China approved the experimental protocol (Protocol code H22049).

### Stimuli and design

**Figure 1:**
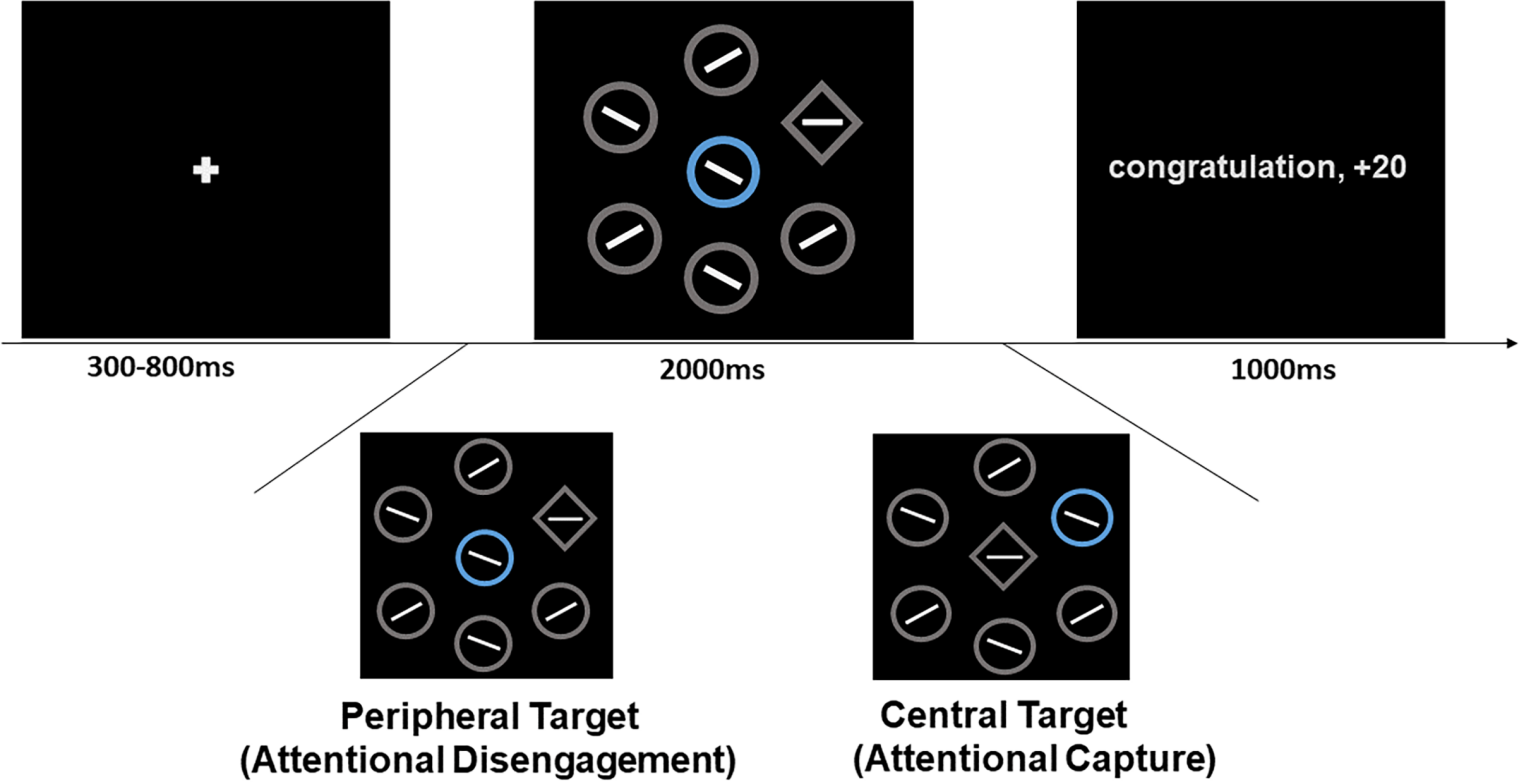
Illustration of the experimental procedure. Sample trial sequences for the visual search. A gray diamond is target, participants’ task is to search target and judge the line segment within target as soon as quickly. If distractor was presented at central location and target was present at periphery, participants had to disengage attention from distractor and search target; if distractor was presented at peripheral location and target was presented at central location, participant need to had to central target. The distractor was imbued with different colours. The blue and orange distractor respectively signalled reward and loss and these colour-outcome relationships were counterbalanced between participants. Especially green distractor and no-distractor signalled neutral outcomes.

We used E-Prime software (Version 2.0) to control visual stimulus presented on an 18.5-inch display screen, with 1920 × 1080 pixel resolution and a 60 Hz refresh rate. We modified the visual search task that based on the study by Watson et al. (2020). Trials began with the presentation of a white cross (0.5° visual angle) for 300-800 ms. Then, a search display was presented for 2,000 ms. The search display consisted of a series of seven shapes (2.0° × 2.0° visual angle), of which only one was a diamond (the target) and the other six were circles. Specifically, there were two ways to present the location of the target at the central fixation location. One way was central-target, where the target was presented at the central display and a singleton distractor was presented at the peripheral location. The other way was peripheral-target, where a singleton distractor was presented at the central display and the target was presented at the peripheral location. Five circles and a diamond target (peripheral-target), or a singleton distractor (central-target) were equally distributed and formed a circular ring with a diameter of 10 degrees of visual angle. The location of diamond or distractor was randomly presented at one of six peripheral locations in each trial. There was a line segment inside each shape. The orientation of line segment was 45° or 135° in each circle and was 0° or 90° in target (see Fig. 1). On each trial, one of circles was rendered in blue (RGB: 0, 192, 255), orange (RGB: 255, 192, 0) or green (RGB: 19, 176, 8). Target and non-singleton were gray (RGB: 188, 188, 188).

There were one practice block and six normal blocks in this experiment. Each normal block consisted of one hundred sixty trials: eighty trials were central-target and eighty trials were peripheral-target. For the central-target type, a quarter of the trials were associated with reward, a quarter with loss, a quarter with neutral, and a quarter with no distractor. The same rule was also applied to the peripheral-target type. There were forty trials in a practice block. Only completion of 60% accuracy in the practice block allowed participants to continue to the normal blocks. At the end of each block, the cumulative scores of the search task were presented.

Differently colored distractors signaled different values. The blue distractor signaled reward: the feedback screen would present “Congratulations, +20 points” for a correct response and “ +2 points” for an erroneous response (an incorrect response or a correct response time higher than 2s), which lasted 1,000 ms. The orange distractor signaled loss: the feedback screen would present “ −1 points” for a correct response and “Sorry, −10 points” for an erroneous response. The colors of blue and orange were balanced among the participants. The green distractor and no-distractor condition signaled no value: the feedback presented “ ± 0 point” according to correct and erroneous responses.

### Procedure

Participants were instructed to perform experiments in a separate room with the same sound insulation and illumination intensity. They were informed that monetary compensation was dependent on their final total points, and to report the orientation of the line segment in the diamond by pressing the F and T keys for horizontally and vertically oriented lines, respectively.

Half of the participants were informed that it was a rewarding trial when a blue circle appeared, and a lost trial when an orange circle appeared (the colored circle was counterbalanced across participants). The mapping reversed for the other half of the participants. In rewarding trials, “if you judge the orientation of the line in the target to be correct within 2s, you will receive 20 points, otherwise you will only receive 2 points”. In lost trials, “if you judge the orientation of the line in the target to be correct within 2s, you will lose 1 point, otherwise you will lose 10 points”. Participants were also informed that it was a no-value trial when a green circle and all gray circles (no-distractor condition) appeared, that is, “you will receive 0 points irrespective of a correct or incorrect response”. The participants were allowed to take a break after finishing one block.

## Results

**Figure 2:**
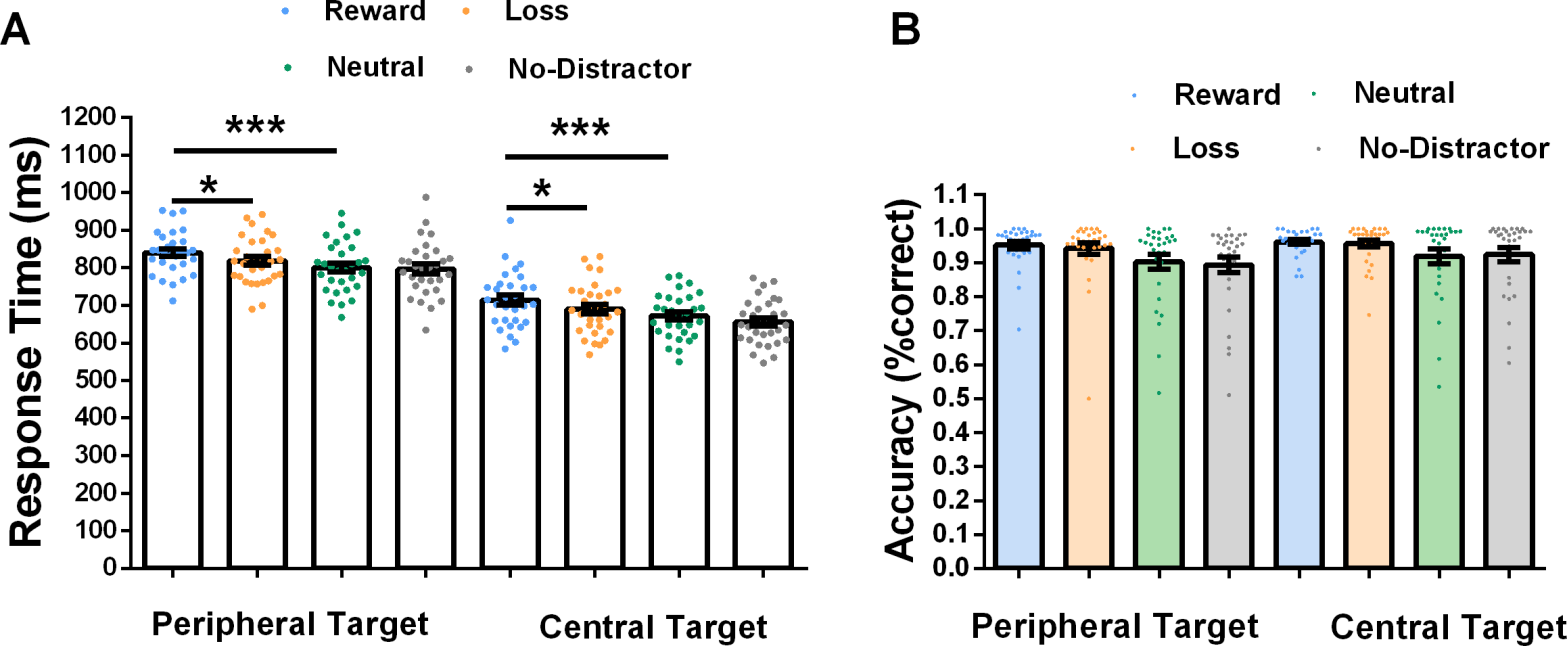
Experiment results. (A) The response time is shown as a function of type of target location (centre, periphery) and value type (rewards, losses, neutral and no-distractor). Each participant’s data points are superimposed. (B) Response accuracy. Error bars represent within-subject standard errors of the mean (*p < .05, ***p < .001).

We have excluded error trials and the response time was faster than 150 ms or slower than 1,100 ms and performed a 2 (location of target: centre and periphery) × 4 (distractor type: reward, loss, neutral and no-distractor) repeated-measures ANOVA on the RTs. The results showed a main effect for location (*F* (1, 29) = 210.81, *p* < .001, *η*^2^ = .73), with faster RTs in the central-target condition (M = 682 ms) than in the peripheral-target condition (M = 814ms). A main effect for distractor type was also significant (*F* (3, 87) = 21.42, *p* < .001, *η*^2^ = .42). The interaction between these two factors was significant (*F* (3, 87) = 3.09, *p* = .03, *η*^2^ = .10). *Post hoc* tests revealed that for peripheral-target type the RTs on reward-distractor (reward: *M* = 840 ms, loss: *M* = 818 ms, neutral: *M* = 800 ms, no-distractor = 796 ms) were significantly larger than those on loss-distractor (*t* = 2.89, *p* = .02) and neutral-distractor trials (*t* = 5.38, *p* < .001), and the RTs on loss-distractor were slower than those on neutral-distractor trials (*t* = 2.48, *p* = .05). There was no significant difference in RTs between neutral-distractor and no-distractor trials (*t* = .54, *p* = .58, all *p* value corrected by Holm method). This results showed that disengagement from a reward-distractor took more time than that from a loss- or neutral-distractor, see Fig. 2A. For central-target type, the RTs on reward-distractor (reward: *M* = 714 ms, loss: *M* = 690 ms, neutral: *M* = 671 ms, no-distractor = 655 ms) were significantly higher than those on loss-distractor (*t* = 3.25, *p* = .01) neutral-distractor (*t* = 5.75, *p* < .001), and the RTs on loss were slower than those on neutral-distractor trials (*t* = 2.49, *p* = .05). The RTs between neutral-distractor and no-distractor showed a marginally significant difference (*t* = 2.19, *p* = .06, all *p* value corrected by Holm method).This results suggested that participants’ attention was captured more in reward-distractor than in loss-distractor and neutral-distractor condition, see Fig. 2A.

The accuracy results revealed that there was a main effect of location (*F* (1, 29) = 7.75, *p* = .01, *η*^2^ = .21), with the accuracy of location in the capture condition being larger than in the disengagement condition (peripheral-target: M = 92%, central-target: M = 94%). There was also a main effect of value distractor (*F* (3, 87) =7.39, *p* < .001, *η*^2^ = .20), (reward: *M* = 96%, loss: *M* = 95%, neutral: *M* = 91%, no-distractor: *M* = 91%). The interaction between location and distractor type was not significant (*F* (3, 87) =1.56, *p* = .20, *η*^2^ = .05), see Fig. 2B.

### Additional analyses

Data analysis was performed in R statistical software, Version 4.3.0 (R Core Team, 2016). To compare the performance in each condition for each participant in visual search tasks, we conducted linear mixed models, using the lme4 package (Bates et al., 2015) and the lmerTest package (Kuznetsova, Brockhoff & Christensen, 2016), with restricted maximum likelihood estimation (REML). Models included fixed effects and random effects. The fixed effect were the effects of value and target location and their interaction effects, and the random effect were the variation in intercept within subjects and the variation in intercept within target-distractor location (there were six locations; a distractor was randomly presented at one of six peripheral location when target was at central fixation, and a target was randomly presented at one of six peripheral locations when distractor was at central fixation) as follows:

RT ∼ value* target location + (1 | subject) + (1 | target-distractor location).

We tried to improve this model by adding value and target location slope within subjects, but none of these models performed better than this model (REML:295036). ANOVA analysis of this model revealed that a main effect of target location (*F* (1, 23283) = 5707.26, *p* < .001) and a main effect of value (*F* (3, 23280) = 134.14, *p* < .001), as well as a significant interaction between them (*F* (3, 23280) = 3.24, *p* = .02). Follow-up analyses of this interaction showed that, for peripheral-target condition the RTs on reward-distractor were notably higher than those on loss-distractor (*t* = 5.68, *p* < .001) and neutral-distractor trials (*t* = 9.78, *p* < .001), and the RTs on loss-distractor were notably slower than neutral-distractor trials (*t* = 4.11, *p* < .001). There was no significant difference in RTs between neutral-distractor and no-distractor (*t* = 1.06, *p* = .28, all *p* value corrected by FDR method). For central-target type, the RTs on reward-distractor were significantly higher than those on loss-distractor (*t* = 7.03, *p* < .001) neutral-distractor (*t* = 11.51, *p* < .001), and the RTs on loss were significantly slower than those on neutral-distractor trials (*t* = 4.50, *p* < .001). There was a significant difference in RTs between neutral-distractor and no-distractor (*t* = 4.36, *p* < .001, all *p* value corrected by FDR method).

ACC ∼ value* target location + (1 | subject) + (1 | target-distractor location).

We analyzed the ACC (accuracy rate) data that was based on this model. ANOVA analysis of this model revealed that a main effect of value (*F* (3, 24,890) = 171.91, *p* < .001) and a main effect of target location (*F* (1, 24,893) = 5,304.98, *p* < .001). There was no interaction of value and target location (*F* (3, 24,889) = 1.57, p = .19).

Results from linear mixed models were consistent with those from previous analysis, suggesting that participants took more time to disengage from a reward-distractor than from a loss- and neutral-distractor, and their attention was more captivated by the reward-distractor than by the loss- and neutral-distractor conditions.

## Discussion

The current study investigated the influences of evaluative stimuli on attentional capture and disengagement. We modified previous visual search paradigm to measure attentional capture and disengagement by manipulating location of target and distractor. It reflects attentional capture when a singleton distractor was presented at periphery and target was presented at central location after fixation disappeared. As attention was already at the centre when the target was shown, it was not necessary to direct attention to the peripheral distractor. The results showed that subjects’ response to central-target was slower when peripheral distractor was associated with reward compared to loss and neutral value, implying that participants’ attention was captured by peripheral reward distractor more than loss and neutral distractor condition. Additionally, it reflects attentional disengagement when a singleton distractor was presented at central location and target was present at periphery, as participants had to shift their attention to peripheral target. The findings indicated that participants took longer to respond to the central-distractor when it was associated with reward than with loss or neutral value, which is in line with previous research (Yan et al., 2022). This could be explained by the fact that positive and negative outcomes can affect the attentional processes, as reward outcomes are linked to the dopaminergic system and the avoidance of loss is associated with the serotonergic pathway, which have different impacts on the degree of attentional capture and disengagement (Gueguen et al., 2021).

Previous studies have found that there was no significant difference in attentional capture between reward- and loss-related trials, which is not in line with our findings (Wang, Yu & Zhou, 2013; Wentura, Muller & Rothermund, 2014). This discrepancy may be due to our manipulation of rewards and losses, which was different from previous studies. We made rewards twice as large in magnitude as losses (Bogdanov et al., 2022; Proudfit, 2015), based on the value bias proposed by prospect theory (Tversky & Kahneman, 1981; Tversky & Kahneman, 1992). Wentura, Muller & Rothermund (2014) compared the effects of reward- and loss-associated distractors when the quantity was equal, while Wang, Yu & Zhou (2013) manipulated the quantity of losses to be larger than rewards. Additionally, previous researchers used a two-phase paradigm, with a valence-induction training phase followed by an unrewarded additional-singleton test phase (where the value-stimuli association had been established in the training phase). Wang, Yu & Zhou (2013) used a visual search task during a test instead of an additional-singleton. These differences may explain the different results.

Studies have demonstrated that people with addiction and anxiety disorders have an abnormal attentional bias towards reward-related stimuli and difficulty shifting away from negative-related stimuli (Anderson, 2019; Rudaizky, Basanovic & MacLeod, 2014; Watson et al., 2019b). Especially, excessive attention to reward-related distractors is associated with drug addiction (Anderson et al., 2013), impulsive behaviors (Albertella et al., 2019), and HIV + patients with a history of risk-taking behavior (Anderson et al., 2016), whereas insufficient attention to reward-related stimuli is associated with attention-deficit hyperactivity disorder (ADHD) and depression (Anderson et al., 2014; Sali et al., 2018). Current studies have only shown that healthy individuals have an attentional bias towards positive reward-distractors more than negative loss-distractors, and they find it difficult to disengage from positive-related distractors relative to negative-related distractors. Future research can expand the scope of sample selection, including unhealthy individuals such as patients with anxiety disorders and depression. We consider that unhealthy individuals with anxiety disorders will show a different pattern in attentional capture and disengagement compared to healthy individuals.

This study has certain limitations. For instance, we did not take intertrial location priming into account. Recent research has shown that the target location from the previous trial can affect the deployment of visual attention, which is known as intertrial location priming (Talcott & Gaspelin, 2020). As we manipulated half of the trials to be central-target trials and the other half to be peripheral-target trials, there is a possibility of intertrial location priming effect that could speed up response-level decisions for targets at central fixation. Additionally, the results of attentional capture and disengagement by evaluative distractors are only at the behavioral level. Although this study suggested that reward-related distractors produced faster reaction times than loss-related distractors in attentional capture and disengagement processes, ERP techniques could provide more insight into the distinct mechanisms of both attentional capture and attentional disengagement when they are modulated by evaluative stimuli.

## Conclusion

In summary, our study can effectively distinguish the processes of attentional capture and attentional disengagement, and highlight the role of evaluative stimuli on these processes. Specifically, we observed that a reward distractor can capture more attention than a loss distractor, and people have difficulty disengaging attention from a reward distractor.

## Additional Information and Declarations

### Data Availability

The following information was supplied regarding data availability: Raw data are available at OSF: Bigbaby. 2023. “Attentional Capture and Disengagement”. OSF. February 15. https://osf.io/jsye3/.
